# Adaptive strategies for the deployment of rapid diagnostic tests for COVID-19: a modelling study

**DOI:** 10.12688/gatesopenres.14202.1

**Published:** 2023-01-27

**Authors:** Lucia Cilloni, Emily Kendall, David Dowdy, Nimalan Arinaminpathy

**Affiliations:** 1 Department of Epidemiology, Johns Hopkins Bloomberg School of Public Health, Baltimore, MD, USA; 2 MRC Center for Global Infectious Disease Analysis, School of Public Health, Imperial College London, London, UK

**Keywords:** COVID-19, lateral flow assays, mathematical modelling

## Abstract

**Background:** Lateral flow assays (LFAs) for the rapid detection of severe acute respiratory syndrome coronavirus 2 (SARS-CoV-2) provide an affordable, rapid and decentralised means for diagnosing coronavirus disease 2019 (COVID-19). Concentrating on urban areas in low- and middle-income countries, we examined whether ‘dynamic’ screening algorithms, that adjust the use of confirmatory polymerase chain reaction (PCR) testing based on epidemiological conditions, could reduce cost without substantially reducing the impact of testing.

**Methods:** Concentrating on a hypothetical ‘second wave’ of COVID-19 in India, we modelled the potential impact of testing 0.5% of the population per day at random with LFA, regardless of symptom status. We considered dynamic testing strategies where LFA positive cases are only confirmed with PCR when LFA positivity rates are below a given threshold (relative to the peak positive rate at the height of the epidemic wave), compared to confirming either all positive LFA results or confirming no results. Benefit was estimated based on cumulative incidence of infection, and resource requirements, based on the cumulative number of PCR tests used and the cumulative number of unnecessary isolations.

**Results:** A dynamic strategy of discontinuing PCR confirmation when LFA positivity exceeded 50% of the peak positivity rate in an unmitigated epidemic would achieve comparable impact to one employing PCR confirmation throughout (9.2% of cumulative cases averted vs 9.8%), while requiring 35% as many PCR tests. However, the dynamic testing strategy would increase the number of false-positive test results substantially, from 0.07% of the population to 1.1%.

**Conclusions:** Dynamic diagnostic strategies that adjust to epidemic conditions could help maximise the impact of testing at a given cost. Generally, dynamic strategies reduce the number of confirmatory PCR tests needed, but increase the number of unnecessary isolations. Optimal strategies will depend on whether greater priority is placed on limiting confirmatory testing or false-positive diagnoses.

## Introduction

Diagnosis has been a critical component of the global coronavirus disease 2019 (COVID-19) response ^1^. Although molecular tests such as reverse-transcriptase polymerase chain reaction (RT-PCR) are highly sensitive ^2^, they are costly and require training to operate. These factors have limited RT-PCR use in low-and-middle-income countries (LMICs) ^3^. Rapid diagnostic tests (RDTs), employing lateral flow assays, offer a more affordable approach to detecting SARS-CoV-2, and have been used widely for community-level testing, as well as for self-testing in households, including in LMICs ^4– 6^. RDTs are, however, less sensitive and specific than PCR ^7^.

In previous work ^8^, we examined the use of RDTs in pandemic response, using modelling to compare different scenarios for their use in cities such as New Delhi, India, and Kampala, Uganda. The results of that analysis suggested that LFAs would affect transmission most efficiently if they were focused on testing symptomatic patients presenting to healthcare facilities, rather than additionally aiming to reach asymptomatic and presymptomatic cases in the community. However, a limitation of that work is that diagnostic algorithms involving LFAs were assumed to remain uniform through time, regardless of the prevalence of SARS-CoV-2. In practice, strategies that can adapt during the course of a pandemic wave – for example, switching to more simplified, rapid algorithms as prevalence increases – may provide an approach for maximising the benefit of LFAs. Here, we sought to examine such strategies using modelling, focusing on the potential impact of dynamic testing strategies during an epidemic consistent with India’s second wave of COVID-19. Although the severity of COVID-19 as a pandemic threat has diminished since 2020, these questions remain relevant for future pandemic response.

## Methods

### Model outline

**Figure 1.**
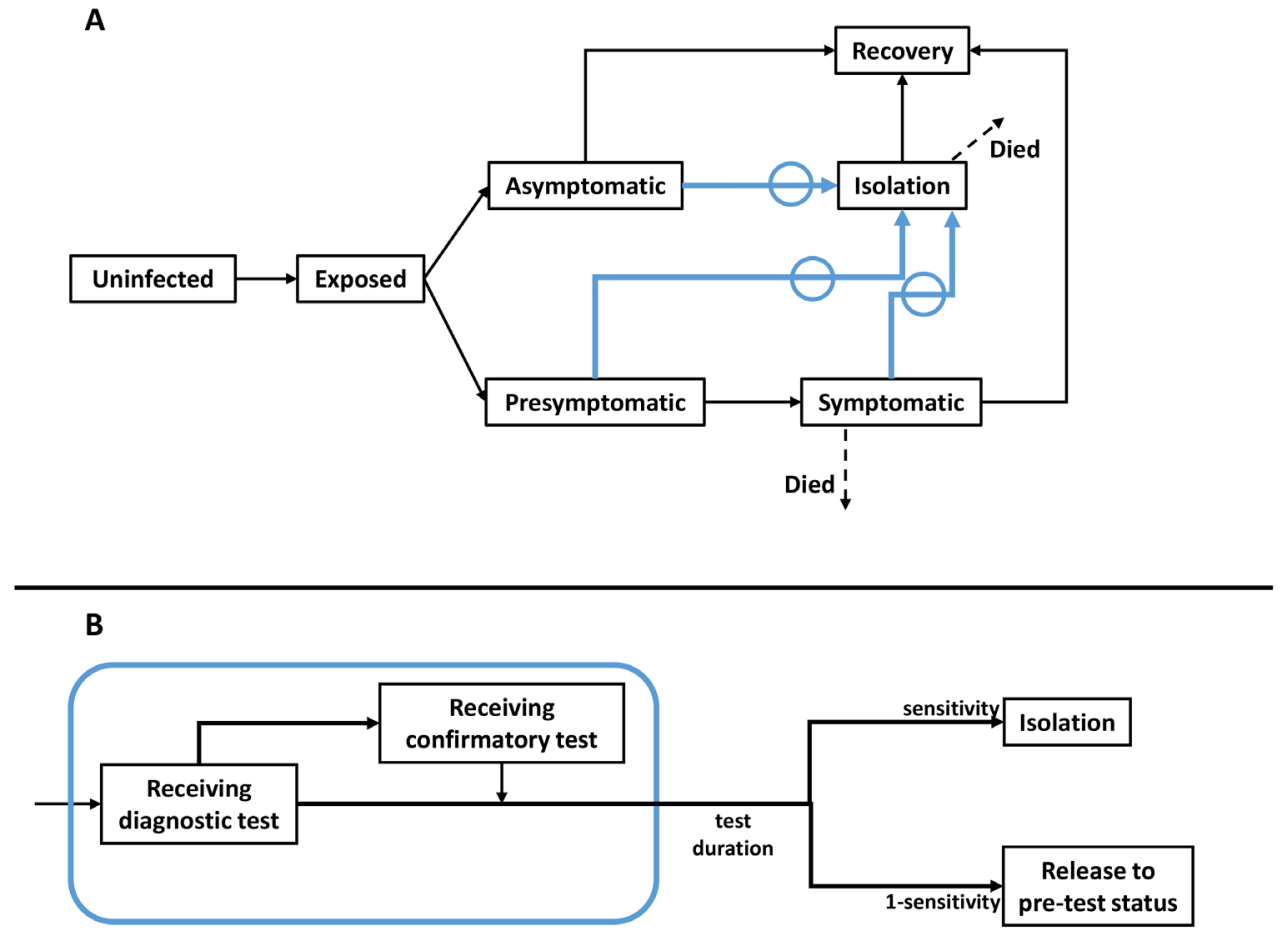
Schematic illustration of the model structure. ( **A**) Compartments representing natural history of SARS-CoV-2, and processes involved in a test-and-isolate intervention. This structure is stratified into three age groups: children (≤ 19 years old), adults (20 – 64 years old), and older adults (≥ 65 years old). As described in the main text, we assume that asymptomatic and pre-symptomatic individuals are infectious, but potentially to a lesser extent than symptomatic cases. Arrows in blue show isolation through testing, shown in greater detail in the bottom panel. ( **B**) Detail of diagnostic testing. We assumed that there is no constraint on the number of LFA tests that can be performed per unit time. To reflect limits on PCR availability in LMICs in a simple way, we assumed that PCR results are only available after three days, and further that individuals are not required to self-isolate during this period.

We built on a deterministic, compartmental model of a hypothetical second wave of SARS-CoV-2 transmission in New Delhi, originally developed in 8, with initial conditions and basic reproduction number similar to those relating to the delta wave in India. The overall model structure is illustrated schematically in Figure 1. Briefly, to account for age-dependent severity of infection, and to capture the population structure in New Delhi, the model incorporates three different age groups: <19 years old, 19 – 64 years old, and 65 years old and above. It also captures important features of the natural history of SARS-CoV-2 infection, including presymptomatic infection (cases prior to developing symptoms) and asymptomatic infection (cases who never develop symptoms), both of which are capable of transmission ^9, 10^. We did not model vaccination, because vaccination coverage had not yet reached substantial levels during the first wave in India ^11^. Although the originally published model ^8^ incorporated additional structure for delays in PCR testing, for simplicity we did not incorporate that structure in the present work. Instead, for the current analysis, we modelled challenges in the availability of PCR in LMIC settings by assuming that the delay for PCR confirmatory testing is fixed at three days (amounting to half of the infectious period) at all stages of the pandemic, and that when PCR confirmatory testing is used, isolations are deferred until LFA positive results are confirmed. As described below, we estimated the number of PCR tests that would be needed under different testing strategies, treating this indicator as a quantity to be minimised.

Model parameters were specified as follows: natural history parameters were drawn from the literature, including estimates of the relative infectivity of asymptomatic/pre-symptomatic vs symptomatic infection. The rate of infectivity per symptomatic case was calibrated in order to yield a basic reproduction number, *R*_0_, of 2.5, consistent with previous work ^12^. Finally, we drew from the literature for the sensitivity and specificity of LFAs and PCR ^2, 3^. See Table S1 in the *Underlying data*, for a full list of model parameters and values ^13^. The code itself is available from GitHub and archived with Zenodo ^14^.

### Scenarios modelled

We concentrated on community-level testing, which aims to use LFAs to identify infectious cases of SARS-CoV-2, regardless of symptom status, and to isolate all who test positive. Previous analysis ^8^ illustrated that a major limitation of such a strategy is that it would lead to a prohibitive number of false-positive diagnoses: it would be critical to implement confirmatory testing, for example using PCR, following any LFA positive test results. We therefore examined whether there would be stages in a pandemic wave when the requirement to confirm positive LFA results could be lifted, in order to reduce costs and minimise any delays in the isolation of individuals with SARS-CoV-2.

The proportion of LFA results that were positive (an indicator of current disease burden that would be readily available in a public testing program, although not in at-home testing) was used to trigger switching between confirmatory testing and LFA alone in the dynamic strategies we evaluated. We determined the peak LFA positivity rate in our model of an unmitigated epidemic wave (i.e., with no isolation of infected individuals), and we defined thresholds relative to this “peak value”.

We modelled the following scenarios:

(i) A ‘low threshold’ dynamic strategy where PCR is used for confirmation of LFA-positive results as long as the proportion of LFA positive tests is less than 10% of the peak value. We assumed that, for LFA positivity rates above this threshold, all individuals testing positive on LFA would be asked to isolate without need for PCR confirmation.
(ii) A ‘medium threshold’ dynamic strategy: the same as (i), but with the LFA positivity threshold set at 50% of the peak value.
(iii) A ‘high threshold’ dynamic strategy: the same as (i), but with the LFA positivity threshold set at 90% of the peak value.
(iv) ‘LFA only’: A non-dynamic strategy where PCR confirmation is never used for LFA-positive individuals, and
(v) ‘LFA+PCR’: A non-dynamic strategy where PCR confirmation is always required for LFA-positive individuals.

The latter two scenarios were included for reference; they represent, respectively, a strategy that would create large numbers of false-positive diagnoses (strategy iv), and one that would involve maximum PCR usage (strategy v).

For each strategy we estimated the daily and cumulative incidence of symptomatic COVID as a measure of epidemiological impact, assuming that the testing regime was initiated before the onset of the pandemic wave. We also calculated two proxies for trade-offs between impact and resource requirements: number of cases averted per PCR test used, and number of cases averted per unnecessary (false-positive) isolation.

### Uncertainty

All parameters were subject to the uncertainty intervals shown in Table S1 in the supporting information. Uncertainty was estimated by using Latin Hypercube Sampling to obtain 250 samples of model parameters; identifying the value of *β* that yielded *R*_0_ = 2.5 for each parameter set; and simulating model projections using each of these 250 samples. Uncertainty in model outputs was calculated using 2.5 ^th^ and 97.5 ^th^ percentiles as the lower and upper 95% uncertainty intervals, while central estimates were obtained using the 50 ^th^ percentile.

All analyses were performed in MATLAB, R2022a. An open-source alternative that may be able to perform similar functions required to repeat this study is GNU Octave.

## Results

**Figure 2.**
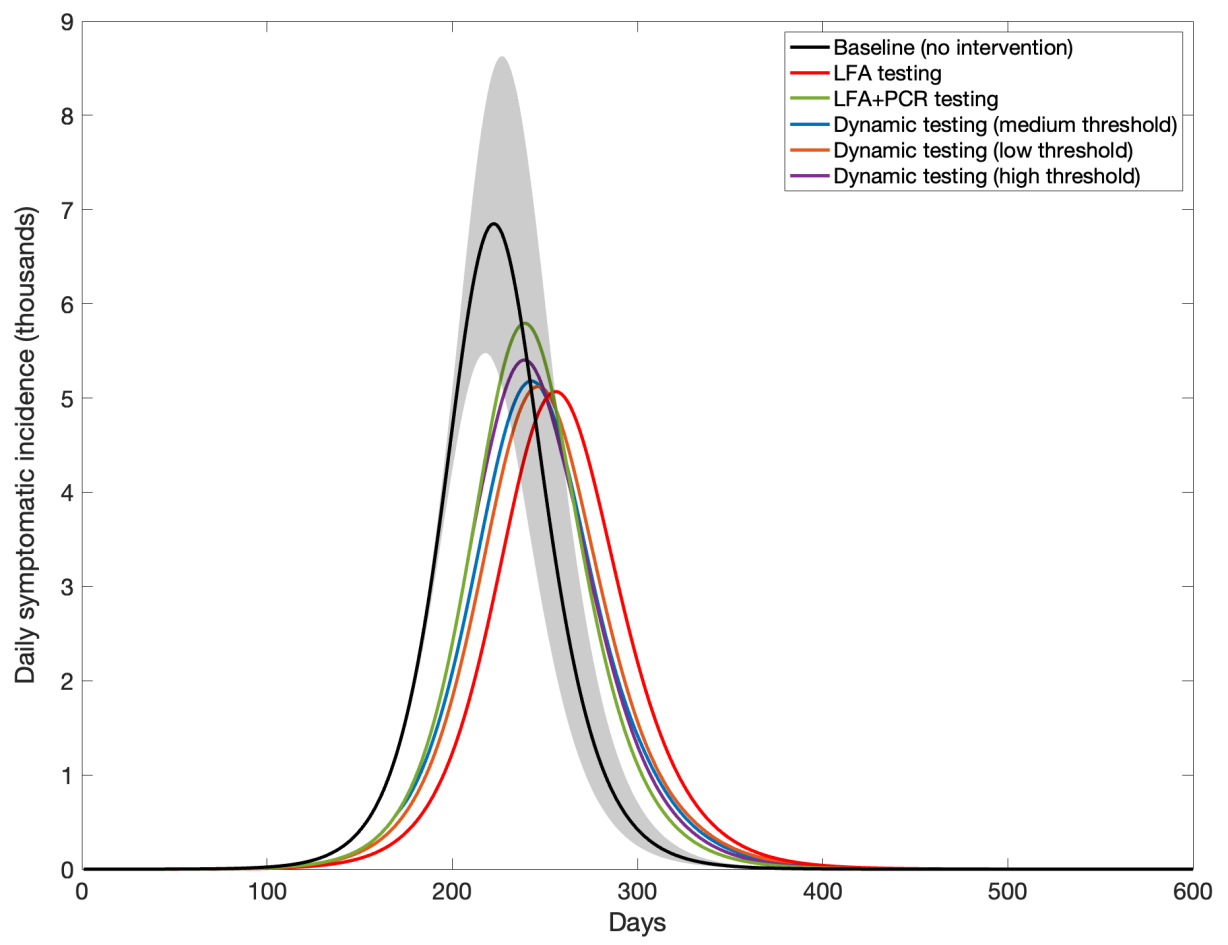
Simulated daily incidence under different LFA scenarios. Curves show simulations consistent with the ‘second wave’ of COVID-19 in India, under the following testing scenarios: ‘LFA testing’ denotes the sole use of LFA for testing with no follow-up confirmation; ‘LFA+PCR’ denotes the use of PCR to confirm LFA-positive results; ‘Dynamic testing (medium threshold)’ denotes PCR confirmation of LFA-positives as long as LFA positivity results are below 50% of peak positivity in an unmitigated wave (no confirmation otherwise); and ‘low’ and ‘high’ thresholds correspond respectively to 10% and 90%. In all scenarios we assumed that a proportion 0.5% of the population is tested with LFA, at random each day, regardless of symptoms, and that all diagnosed with SARS-CoV-2 are isolated. Solid lines show central estimates, and shaded areas show 95% uncertainty intervals (for clarity, only shown illustratively in the baseline scenario).

Figure 2 shows the results of each community-level testing strategy on the hypothetical second epidemic wave of COVID-19 in India, with overall cases averted shown in Table 1. For example, an intervention testing only with LFA, with isolation of all LFA-positive individuals and no need for confirmation of positive LFA results, would avert 9.8% (95% CrI 6.5 – 13.2%) of symptomatic cases, while requiring PCR confirmation for all LFA-positive individuals would reduce this impact by about one-third, to 6% (95% CrI 4 – 8%). Each of the dynamic strategies was projected to have an epidemiological impact intermediate to these two extremes.

**Table 1.** Summary of epidemiological impact and resource use. Entries show values summarising the outcomes in Figure 2 – Figure 3.

	Incidence reduction (compared to baseline scenario) [95% CrI]	PCR consumption	Unnecessary isolations (relative to population size)
**Dynamic (medium ** **threshold)**	9.20% [6.14%-12.36%]	1.8 million	1.10%
**Dynamic model ** **(low threshold)**	9.70% [6.23%-13%]	830,000	1.70%
**Dynamic model ** **(high threshold)**	7.71% [5.28%-10.64%]	1.76 million	0.45%

**Figure 3.**
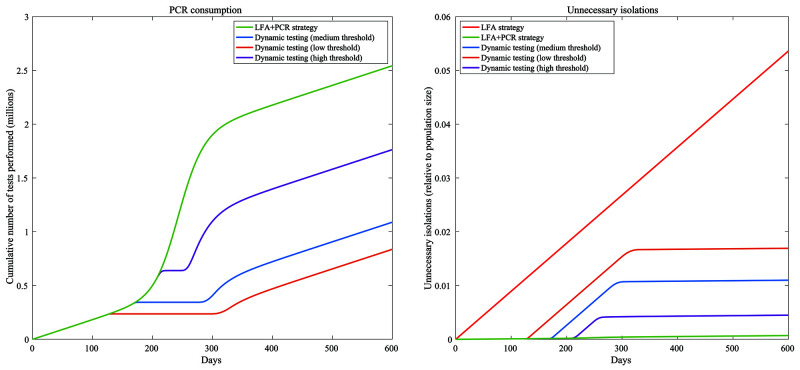
Proxies for costs of different testing strategies. The left-hand panel shows the cumulative number of PCR tests used over time, while the right-hand panel shows the cumulative number of unnecessary isolations over time (arising from false-positive diagnoses of SARS-CoV-2), as a proportion of the population.

Figure 3 shows two proxies of resource requirement, namely PCR test volume and unnecessary isolations. An LFA+PCR strategy requires the greatest number of PCR tests (green curve, left-hand panel), while also incurring the fewest unnecessary isolations (right-hand panel). On the other hand, while an LFA-only strategy naturally incurs no PCR usage, it also leads to over 5% of the population being unnecessarily isolated (red curve, right-hand panel). In both cases, dynamic strategies can mitigate these costs substantially. For example, a medium-threshold strategy, one that requires PCR confirmation only when the proportion of LFA positivity is below 50% of peak positivity in an unmitigated wave, would require a total of 1.8 million PCR tests (compared to 2.5 million for an LFA+PCR strategy), and would incur unnecessary isolations for 1.1% of the population.

**Figure 4.**
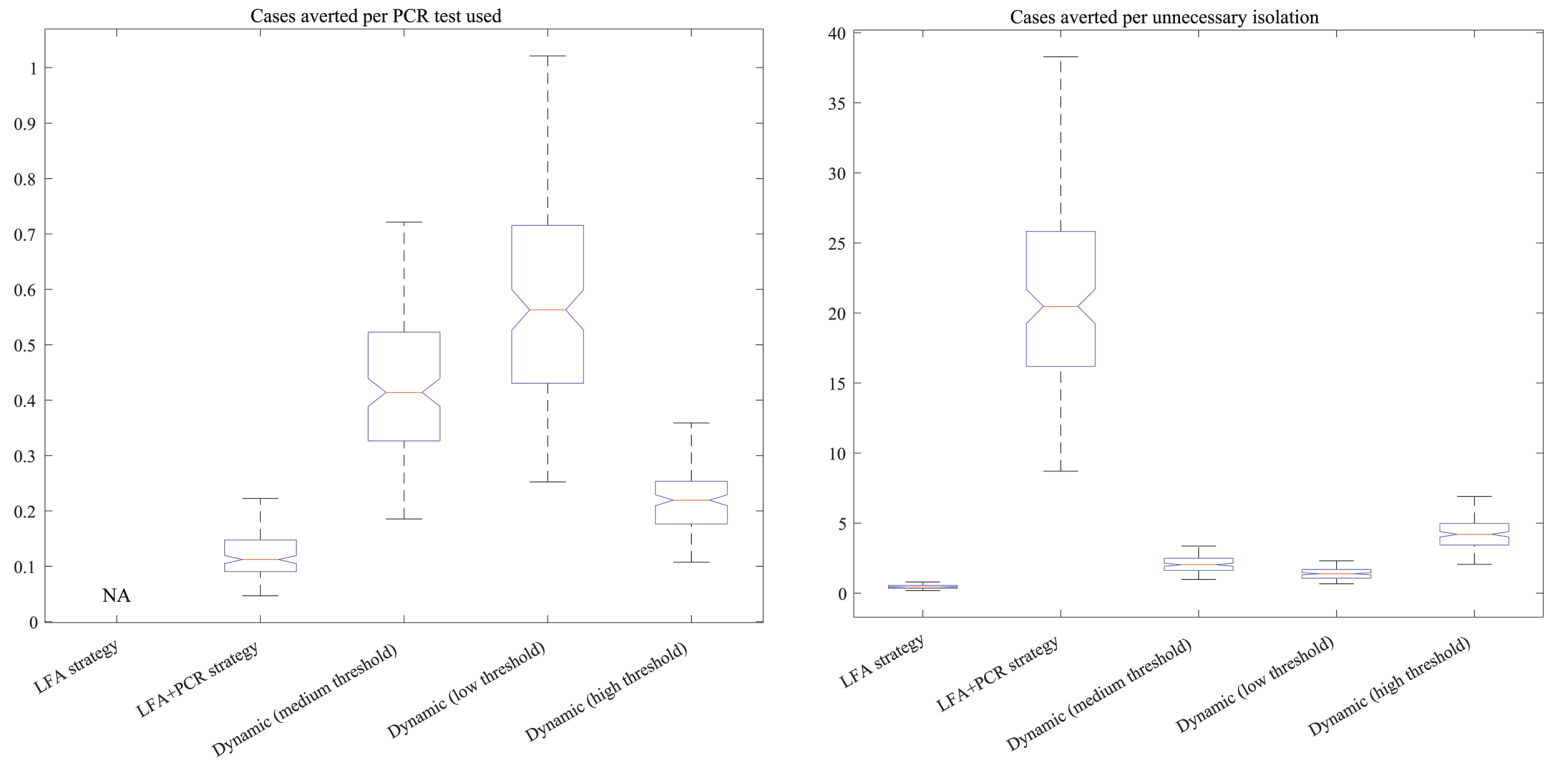
Proxies for incremental cost-effectiveness ratios (ICERs) under the different testing strategies. As a measure of health gains for the denominator for ICER calculations, we estimated the symptomatic cases averted relative to a scenario of no community-level LFA testing. In each plot, the horizontal red line shows median estimates; the upper and lower edges of the blue polygons show 2.5 ^th^ and 97.5 ^th^ percentiles; and the upper and lower ‘whiskers’ show the extreme values. The left-hand panel shows ICERs in terms of cases averted per PCR (polymerase chain reaction) test used, while the right-hand plot shows them in terms of cases averted per unnecessary isolation. Strategies shown are as follows. LFA: using LFA (lateral flow assay) only, with no PCR confirmation. LFA+PCR: confirming all LFA-positive results with PCR. Dynamic: Strategies where the need for PCR confirmation of LFA-positives is lifted when LFA test positivity exceeds a given threshold, here showing ‘low’, ‘medium’ and ‘high’ thresholds as described in the main text.

Figure 4 compares strategies in terms of the cases averted per PCR test used (left-hand panel), and cases averted per unnecessary isolation (right-hand panel). In both cases, higher values correspond to more favourable strategies. Results echo the overall trade-off shown in Figure 3: that in general, strategies that are favourable in terms of cases averted per PCR test used are less favourable in terms of cases averted per unnecessary isolation. Quantitative estimates behind these results are listed in Table 1.

## Discussion

In pandemic response, rapid testing at the community level might offer important opportunities to reduce transmission, but only if there are ways to mitigate false-positive results within the bounds of health system constraints. Building on previous work, our analysis illustrates that some options for doing so could be provided by dynamic strategies for the use of PCR testing for confirmation of positive LFA results. In particular, strategies that impose a threshold for LFA positivity, above which PCR confirmation of positive LFA results is no longer necessary, can offer a compromise between the large number of PCR tests required when confirming all LFA positives with PCR, and the large number of unnecessary isolations when using LFA alone ( Figure 3 and Figure 4).

In any given setting, the specific choice of threshold will depend on the local conditions and constraints; in particular, a key consideration is whether PCR capacity is a more pressing constraint than the need to avoid unnecessary isolations. Where PCR capacity is tightly constrained, a more liberal threshold for removing the requirement for PCR confirmation would be favoured. By contrast, where isolation capacity is tightly constrained, this threshold should be more stringent. In order to translate these findings into an appropriate choice of threshold for any given setting, further work would need to combine the costs of PCR usage and unnecessary isolations on a common footing.

We have modelled thresholds depending on the LFA positivity rate at any given point in time. Adapting LFA strategies in response to this rate therefore requires LFA test results to be reported, at least in a representative proportion. While reporting of home-based test results is currently recommended, it is generally not done ^15^. In future, LFAs having the capacity to report test results automatically could improve efforts to monitor the spread of infection and adapt testing strategies accordingly.

As with any modelling analysis, our work has some limitations to note. Our model assumes a simplified, homogenous population structure, whereas in reality, in the first few waves of COVID-19, the spread of SARS-CoV-2 was more extensive in urban slum populations than elsewhere in India ^16^. We also do not model relationships between infectivity and LFA detection. While LFAs are not as sensitive as PCR, there is evidence to suggest that those cases of SARS-CoV-2 that are detectable by LFA are also the most infectious ^6, 17, 18^, and we would expect such variation to increase the epidemiological impact of LFA-only strategies from that estimated here. We only evaluated dynamic strategies that changed the sequence of testing with the probability of a positive test. Other dynamic strategies could also, for example, impose a requirement for isolation while awaiting confirmatory testing, rather than removing the need for a confirmatory test. Finally, we have focused on one example of the use of LFAs and PCR tests: identifying conditions where PCR need not be used to confirm LFA positives. For future work, other possible areas relevant to transmission include the use of LFAs for surveillance during periods of low infection activity, and switching to PCR confirmation of LFA negatives during the epidemic peak. In all cases, limiting the requirement for PCR testing will be an important consideration for LMICs.

In conclusion, rapid tests can play an important role in reducing opportunities for transmission, but their use must be planned carefully in order to avoid undue adverse impacts, either on the population or on the healthcare system. Mathematical modelling can be a helpful tool for weighing these trade-offs, not only for COVID-19, but also for future pandemics.

## Data Availability

Zenodo: Adaptive strategies for the deployment of rapid diagnostic tests for COVID-19: a modelling study. https://doi.org/10.5281/zenodo.7401171 ^13^. This project contains the following information: Model overview Governing equations Model execution Data are available under the terms of the Creative Commons Attribution 4.0 International license (CC-BY 4.0). Source code available from: https://github.com/lmcilloni/covid-RDT Archived source code at time of publication: https://doi.org/10.5281/zenodo.7410262 ^14^ License: GNU General Public License v3.0

## References

[1] Botti-Lodovico Y Rosenberg E Sabeti PC : Testing in a Pandemic — Improving Access, Coordination, and Prioritization. *N Engl J Med.* 2021;384(3):197–199. 10.1056/NEJMp2025173 33472283

[2] Böger B Fachi MM Vilhena RO : Systematic review with meta-analysis of the accuracy of diagnostic tests for COVID-19. *Am J Infect Control.* 2021;49(1):21–29. 10.1016/j.ajic.2020.07.011 32659413 PMC7350782

[3] Peeling RW Olliaro PL Boeras DI : Scaling up COVID-19 rapid antigen tests: promises and challenges. *Lancet Infect Dis.* 2021;21(9):e290–5. 10.1016/S1473-3099(21)00048-7 33636148 PMC7906660

[4] Stohr JJJM Zwart VF Goderski G : Self-testing for the detection of SARS-CoV-2 infection with rapid antigen tests for people with suspected COVID-19 in the community. *Clin Microbiol Infect.* 2022;28(5):695–700. 10.1016/j.cmi.2021.07.039 34363945 PMC8336990

[5] Dinnes J Deeks JJ Berhane S : Rapid, point-of-care antigen and molecular-based tests for diagnosis of SARS-CoV-2 infection. *Cochrane Database Syst Rev.* 2021;3(3):CD013705. 10.1002/14651858.CD013705.pub2 33760236 PMC8078597

[6] Lopera TJ Alzate-Ángel JC Díaz FJ : The Usefulness of Antigen Testing in Predicting Contagiousness in COVID-19. *Microbiol Spectr.* 2022;10(2):e0196221. 10.1128/spectrum.01962-21 35348350 PMC9045251

[7] Krüttgen A Cornelissen CG Dreher M : Comparison of the SARS-CoV-2 Rapid antigen test to the real star Sars-CoV-2 RT PCR kit. *J Virol Methods.* 2021;288:114024. 10.1016/j.jviromet.2020.114024 33227341 PMC7678421

[8] Baik Y Cilloni L Kendall E : Symptom-based vs asymptomatic testing for controlling SARS-CoV-2 transmission in low- and middle-income countries: A modelling analysis. *Epidemics.* 2022;41:100631. 10.1016/j.epidem.2022.100631 36174427 PMC9511882

[9] Huff HV Singh A : Asymptomatic Transmission During the Coronavirus Disease 2019 Pandemic and Implications for Public Health Strategies. *Clin Infect Dis.* 2020;71(10):2752–6. 10.1093/cid/ciaa654 32463076 PMC7314132

[10] Johansson MA Quandelacy TM Kada S : SARS-CoV-2 Transmission From People Without COVID-19 Symptoms. *JAMA Netw Open.* 2021;4(1):e2035057. 10.1001/jamanetworkopen.2020.35057 33410879 PMC7791354

[11] Choudhary OP Choudhary P Singh I : India’s COVID-19 vaccination drive: key challenges and resolutions. *Lancet Infect Dis.* 2021;21(11):1483–1484. 10.1016/S1473-3099(21)00567-3 34529961 PMC8437681

[12] Mandal S Arinaminpathy N Bhargava B : Plausibility of a third wave of COVID-19 in India: A mathematical modelling based analysis. *Indian J Med Res.* 2021;153(5&6):522–532. 10.4103/ijmr.ijmr_1627_21 34643562 PMC8555606

[13] Cilloni L Kendall E Dowdy D : Adaptive strategies for the deployment of rapid diagnostic tests for COVID-19: a modelling study.[Dataset]. *Zenodo.* 2022. 10.5281/zenodo.7401171 PMC12274328 40688813

[14] Cilloni L : lmcilloni/covid-RDT: covid-RDT (covidRDT.v1.2). *Zenodo.* 2022. 10.5281/zenodo.7410262

[15] Rader B Gertz A Iuliano AD : Use of At-Home COVID-19 Tests - United States, August 23, 2021-March 12, 2022. *MMWR Morb Mortal Wkly Rep.* 2022;71(13):489–94. 10.15585/mmwr.mm7113e1 35358168 PMC8979595

[16] Murhekar MV Bhatnagar T Selvaraju S : SARS-CoV-2 antibody seroprevalence in India, August-September, 2020: findings from the second nationwide household serosurvey. *Lancet Glob Heal.* 2021;9(3):e257–e266. 10.1016/S2214-109X(20)30544-1 33515512 PMC7906675

[17] Nordgren J Sharma S Olsson H : SARS-CoV-2 rapid antigen test: High sensitivity to detect infectious virus. *J Clin Virol.* 2021;140:104846. 10.1016/j.jcv.2021.104846 33971580 PMC8105081

[18] Kessler HH Prüller F Hardt M : Identification of contagious SARS-CoV-2 infected individuals by Roche’s Rapid Antigen Test. *Clin Chem Lab Med.* 2022;60(5):778–85. 10.1515/cclm-2021-1276 35258234

