# Supplementary material for: Adaptive strategies for the deployment of rapid diagnostic tests for COVID-19: a modelling study
Source: Gates Open Res. 2025 May 27;7:6. Originally published 2023 Jan 27. [Version 2] doi: 10.12688/gatesopenres.14202.2 (PMC12274328; doi:10.12688/gatesopenres.14202.2)
Supplement: Peer review history

# Reviewer report: Reviewer response for version 2

**DOI:** 10.21956/gatesopenres.17750.r39709

**Published:** 2025-07-18

**Authors:** Naim Che Kamaruddin

**Affiliations:** 1 Universiti Malaya, Kuala Lumpur, Federal Territory of Kuala Lumpur, Malaysia; 2 Clinical Research Centre, Sunway Medical Centre (Ringgold ID: 538534), Bandar Sunway, Selangor, Malaysia

Dear Authors,

 Congratulations on the completion of your study and thank you for sharing your work in this manuscript. I agree the earlier comments, particularly those made by Dr. Lucia, who has outlined several important revisions. I would like to offer the following additional suggestions to further strengthen the manuscript:

 1. To specify the study aim. While the manuscript is generally understandable and the results are adequately presented, the overall readability would benefit from improved sentence structure. I recommend clearly stating the study aim in a concise sentence to support your hypothesis. For example: “To examine and model COVID-19 testing algorithms/strategies to reduce testing costs and minimise unnecessary quarantine or isolation.”

 2. To clarify the vaccination Coverage. In the sentence, “Despite the introduction of the COVID-19 vaccine in January of 2021, the coverage by March was not high enough to effectively curb the delta wave surge,” the term “not high enough” is vague. Please consider specifying the vaccination coverage during the study period. Additionally, clarify whether the delta wave was primarily due to insufficient vaccination coverage, or due to the variant’s high transmissibility or a combination of both.

 3. To define abbreviations. Please define “LFA” (lateral flow assay) upon first use in the introduction (paragraph 3). Please double check for the whole manuscript.

 4. To justify the false positive implications. The discussion around false positives is currently underdeveloped. You mention that the main implication is unnecessary isolation. However, from a public health standpoint, false negatives may have more serious consequences as they could allow for transmission. Please expand your justification regarding the trade-offs between false positives and false negatives, and the broader implications for public health decision-making.

 5. To clarify on the rationale against universal PCR testing. An important question arises, which is why not recommend universal PCR testing for exposed or suspected cases, followed by a short quarantine or isolation for the incubation period (e.g., 3–5 days), and release upon a negative PCR and absence of symptoms? This approach could also be cost-effective and reduce transmission. How does your proposed strategy in this manuscript compare in terms of cost-effectiveness and public health benefit?

 6. To add information on the LFA test. Please include the sensitivity and specificity of the LFA tests used in your model, as these values are critical for interpreting the results.

 7.  To rephrase sentences for clarity. Consider rephrasing this sentence for clarity and connections with your content, “In practice, strategies that can adapt during the course of a pandemic wave – for example, switching to more simplified, rapid algorithms as prevalence increases – may provide an approach for maximising the benefit of LFAs.”

 8. To clarify feferenced model. In the sentence “We built on a deterministic, compartmental model of a hypothetical second wave of SARS-CoV-2 transmission in New Delhi, originally developed in 16,” please clarify what reference “16” refers to. Is this your previous work or an external study?

 9. To define the sample size. How many total individuals were included in the model? How were populations such as uninfected, exposed, asymptomatic, and presymptomatic individuals defined and tracked? I suggest to summarizing key details currently in the supplementary material into the main methodology section for transparency.

 10. To clarify figure/table interpretation. The interpretation of Table 3 and the associated figures is somewhat confusing. You state that LFA+PCR results in the highest PCR test volume and the fewest unnecessary isolations. However, wouldn’t the identification of unnecessary isolations require a confirmatory PCR in all cases? If so, how is unnecessary isolation measured in the LFA-only scenario without PCR confirmation? Please clarify this comparison and the underlying assumptions.

 11. To refine concluding remark. The current conclusion is too general and does not fully reflect the study’s specific findings. I recommend rephrasing it to synthesise your results more directly. For example: Our modelling suggests that strategic implementation of LFA-based algorithms particularly when combined with PCR confirmation can reduce transmission and testing costs during high-prevalence phases, while minimising unnecessary isolation. These findings support the importance of adaptable, evidence-based testing policies for future pandemic preparedness.”

 Thank you again for the opportunity to review this interesting manuscript. I hope these comments help strengthen your final submission.

Is the work clearly and accurately presented and does it cite the current literature?

Partly

If applicable, is the statistical analysis and its interpretation appropriate?

Partly

Are all the source data underlying the results available to ensure full reproducibility?

Partly

Is the study design appropriate and is the work technically sound?

Partly

Are the conclusions drawn adequately supported by the results?

Partly

Are sufficient details of methods and analysis provided to allow replication by others?

No

Reviewer Expertise:

Epidemiology; Infectious Disease Immunology; Tropical Medicine; Respiratory Infections

I confirm that I have read this submission and believe that I have an appropriate level of expertise to confirm that it is of an acceptable scientific standard.

# Reviewer report: Reviewer response for version 2

**DOI:** 10.21956/gatesopenres.17750.r39567

**Published:** 2025-06-12

**Authors:** Flaminia Tomassetti

**Affiliations:** 1 University of Rome Tor Vergata, Rome, Italy

I appreciate the Authors' effort to response partly to my comments; however, I have one last concern.

- Where is Figure 1?
- The Discussion still lacks criticism.

Is the work clearly and accurately presented and does it cite the current literature?

Partly

If applicable, is the statistical analysis and its interpretation appropriate?

Yes

Are all the source data underlying the results available to ensure full reproducibility?

Yes

Is the study design appropriate and is the work technically sound?

Yes

Are the conclusions drawn adequately supported by the results?

Partly

Are sufficient details of methods and analysis provided to allow replication by others?

Yes

Reviewer Expertise:

Clinical Pathology, COVID-19, Clinical Biochemistry, Immunology

I confirm that I have read this submission and believe that I have an appropriate level of expertise to confirm that it is of an acceptable scientific standard, however I have significant reservations, as outlined above.

# Reviewer report: Reviewer response for version 1

**DOI:** 10.21956/gatesopenres.15508.r38537

**Published:** 2025-01-10

**Authors:** Flaminia Tomassetti

**Affiliations:** 1 University of Rome Tor Vergata, Rome, Italy

The manuscript titled “Adaptive strategies for the deployment of rapid diagnostic tests for COVID-19: a modelling study” has the potential to add to managing SARS-CoV-2 spread and could be more interesting if the strategy proposed could fit also for another health emergency.

 However, some vital information is missing in the manuscript as follows:

 Major revision:

 Abstract

1. The aim is not totally clear.
2. Include the threshold value in the Methods

Introduction

1. The introduction is poor, and the references are a little outdated. The Authors should amplify the first paragraph of this section.
2. The Authors should include something about managing the virus spread, the anti-contagion rules and the safety protocols for virus detection in megalopolis, such as New Delhi.

Methods

1. Please include the years of collecting data (2020-21) for the model.
2. Figure 1 is not clear: should it summaries the current and well-known infection and detection of SARS-CoV-2? I think that the Figure is redundant and is not adding anything vital to the text.

Results

1. The Results are well written and the figures/tables support consistently the data.

Discussion

1. The Discussion should be more contextualized to the results obtained in this study.
2. How do the Authors explain the increase of false positive test results?
3. How do the Authors justify their model for the other pandemic wave (Delta/Omicron)?
4. The Authors should also discuss about the benefits/costs of their model.

Is the work clearly and accurately presented and does it cite the current literature?

Partly

If applicable, is the statistical analysis and its interpretation appropriate?

Yes

Are all the source data underlying the results available to ensure full reproducibility?

Yes

Is the study design appropriate and is the work technically sound?

Yes

Are the conclusions drawn adequately supported by the results?

Partly

Are sufficient details of methods and analysis provided to allow replication by others?

Yes

Reviewer Expertise:

Clinical Pathology, COVID-19, Clinical Biochemistry, Immunology

I confirm that I have read this submission and believe that I have an appropriate level of expertise to confirm that it is of an acceptable scientific standard, however I have significant reservations, as outlined above.

# Response

**Published:** 2025-05-12

**Authors:** Lucia Cilloni

**Affiliations:** Johns Hopkins Bloomberg School of Public Health, USA

General comments response:

 We thank the reviewer for their feedback. While the strategy discussed in this paper is specific to the COVID-19 pandemic, we believe the ideas could be generalizable to other infectious diseases as well. We have therefore added the following brief paragraph to the Discussion:

 “Importantly, while we have evaluated the role of LFAs in testing for SARS-CoV-2, the principle of this analysis – evaluating tradeoffs between rapid isolation and reduction of false-positives through confirmatory testing – applies to epidemics of other infectious diseases as well. Often, LFAs aim for speed and high sensitivity but may have insufficient specificity to take definitive action without confirmatory testing. As such, analyses such as this one, in which tradeoffs between speed of testing and the cost of false-positive results, will be relevant as novel LFAs are developed for other infectious diseases.”

 Abstract comments response:

 We have amended the Abstract to include the following text:

 “Concentrating on urban areas in low- and middle-income countries, the aim of this analysis was to estimate the degree to which ‘dynamic’ screening algorithms, that adjust the use of confirmatory polymerase chain reaction (PCR) testing based on epidemiological conditions, could reduce cost without substantially reducing the impact of testing.”

 And:

 “We considered dynamic testing strategies where LFA positive cases are confirmed with PCR when LFA positivity rates are below a given threshold (10%, 50% and 90% of the peak positivity rate at the height of the epidemic wave),…”

 Introduction comments response:

 In response to the comment about the outdated "Introduction" references, similar to the comment from Reviewer 1, we performed a detailed literature review of LFAs for SARS-CoV-2. However, we found that the most-cited references were all from 2022 or earlier. We have added additional references, though noting that these are still from 2021 and before.

 We have added a paragraph to the Introduction about COVID-19 in India, and in New Delhi, at the time of the second wave.

 “In New Delhi, India, during the first wave of the pandemic, the state government implemented a task force in charge of testing, tracing and tracking new infections, with a combination of reverse-transcriptase polymerase chain reaction (RT-PCR) tests, point-of-care molecular tests and rapid antibody tests, in an effort to detect and isolate incident cases of COVID-19 quickly and reduce transmission…”

 We have also added to the Discussion to highlight this point as a limitation of our study: that we did not address the potential impact of other interventions, such as social distancing and vaccination (both of which played a key role in the COVID response in India). Please see p.10,

 “Concentrating on the impact of testing strategies, we do not model the potential impact of other measures such as social distancing and lockdowns or indeed of vaccination. In reality, in the wake of the devastating ‘delta wave’ in India, the rapid rollout of the world’s largest COVID-19 vaccination programme had substantial impact on disease burden.”

 Methods comments response:

 We have added the years of collecting data to the Methods. Regarding Figure 1, this figure aims to outline the compartments that make up the model used for the analysis. It highlights both the natural history processes of SARS-CoV-2 that are accounted for in the modeling, but also the compartments and processes that are involved in the proposed interventions, with the blue arrows and squares highlighting what processes are being changed to assess the different scenarios being considered. While these data are in the text, we feel that the Figure will help readers visualize the model more effectively and would thus argue for its retention.

 Discussion comments response:

 Performing tests in a sequential manner increases the overall testing algorithm’s specificity considerably. Therefore when it is possible to utilize a confirmatory test, such as PCR, this will ensure that true-negatives are appropriately identified, if they get through the first test as false-positives.

 The reason the model focused on the Delta wave in India was because at the time of the analyses the omicron wave was still new and developing. We have added a justification in the Discussion:

 “The model was calibrated to reflect transmission dynamics representative of the delta wave, rather than the more recent omicron wave. At the time of the analysis, the omicron wave was still new and developing. Since the omicron variant was found to be more contagious than the previous ones, we believe we believe our results would show higher epidemiological impact from utilizing LFAs without PCR confirmation in order to isolate infected individuals as quickly as possible.”

 An important benefit of this updated model is its ability to utilize a dynamic LFA positivity threshold to decide whether PCR confirmation is or isn’t necessary. The implications of this are outlined in the Discussion:

 “In particular, our analysis found that strategies that impose a threshold for LFA positivity, above which PCR confirmation of positive LFA results is no longer necessary, can offer a compromise between the large number of PCR tests required when confirming all LFA positives with PCR, and the large number of unnecessary isolations when using LFA alone.”

 We discuss model limitations in the Discussion paragraph, “As with any modelling analysis, our work has some limitations to note…”, noting limitations around model assumptions and on the possible combinations of tools used for diagnosis.

# Reviewer report: Reviewer response for version 1

**DOI:** 10.21956/gatesopenres.15508.r38543

**Published:** 2025-01-03

**Authors:** Cihan Papan

**Affiliations:** 1 University Hospital Bonn, Bonn, Germany

This is modelling study that investigated different testing strategies with regard to SARS-CoV-2 in India, ranging from lateral flow assay tests based on distinct thresholds of positivity to confirmation by polymerase chain reaction of all positive lateral flow assay tests. The manuscript is well written, and the methodology is sound. The assumptions are explained with enough details and reference. Some of the references seem to be older, owing to the fact that manuscript was originally written in 2022/23. Hence, the authors could make an effort to find some more recent references. 

 I have one additional question: I may have misunderstood this, but I am unsure how a LFA only strategy would lead to the highest rate of averted symptomatic cases (you would have the most false positives here) as opposed to the scenario where all positives are PCR confirmed. Maybe an explanation could help.

Is the work clearly and accurately presented and does it cite the current literature?

Partly

If applicable, is the statistical analysis and its interpretation appropriate?

Yes

Are all the source data underlying the results available to ensure full reproducibility?

Yes

Is the study design appropriate and is the work technically sound?

Yes

Are the conclusions drawn adequately supported by the results?

Yes

Are sufficient details of methods and analysis provided to allow replication by others?

Yes

Reviewer Expertise:

infectious diseases epidemiology, diagnostics

I confirm that I have read this submission and believe that I have an appropriate level of expertise to confirm that it is of an acceptable scientific standard.

# Response

**Published:** 2025-05-12

**Authors:** Lucia Cilloni

**Affiliations:** Johns Hopkins Bloomberg School of Public Health, USA

We thank the reviewer for their feedback and comments. In response to the comment about outdated references, we performed a revised review of references – but found that the highest-quality, most-cited references were all from 2022 or earlier, likely owing to the reduction in interest in testing for SARS-CoV-2 after the stabilization of the COVID-19 pandemic. We have added a more recent reference to our introduction.

 Regarding the question about why the LFA strategy averts more cases than the scenario where all LFA-positives also receive PCR, while it is true that this strategy results in a large number of false-positives, it also results in the highest number of true-positives (i.e., loss of specificity in favor of sensitivity). When all individuals who are LFA-positive need to be confirmed with PCR, those individuals experience a delay in isolation while awaiting PCR confirmation. We now clarify in the Methods, under “Scenarios modelled”:

 “While using LFAs alone would lead to rapid isolation of people with SARS-CoV-2 (and thus greater reduction in transmission), previous analysis illustrated that a major limitation of such a strategy is that it would lead to a prohibitive number of false-positive diagnoses. It would therefore be critical to implement confirmatory testing, for example using PCR, following any LFA positive test results.”
